# Phytoplankton Communities and Their Relationship with Environmental Factors in the Waters around Macau

**DOI:** 10.3390/ijerph19137788

**Published:** 2022-06-24

**Authors:** Rui He, Huan Luo, Ning He, Wenlong Chen, Fang Yang, Weijie Huang, Ning Li, Lingling Sun, Songyao Peng

**Affiliations:** 1 Pearl River Hydraulic Research Institute, Guangzhou 510611, China; rui-he@hotmail.com (R.H.); gzluohuan@163.com (H.L.); xinlpingj20@126.com (W.C.); jnuherui@gmail.com (W.H.); lining_10@163.com (N.L.); 15920308037@139.com (L.S.); 2 State Key Laboratory of Organic Geochemistry, Guangzhou 510640, China; 3 Guangdong Provincial Engineering Technology Research Center for Life and Health of River & Lake, Guangzhou 510611, China; 4 School of Life Science and Resources Environment, Yichun University, Yichun 336000, China; hening2010@jxycu.edu.cn

**Keywords:** phytoplankton, community structure, multivariate analysis, Macau

## Abstract

An investigation of the waters around Macau collected 43 phytoplankton species belonging to 29 genera and 5 phyla, including 32 species from 22 genera of Bacillariophyta, 7 species from 3 genera of Pyrrophyta, 2 species from 2 genera of Cyanophyta, and 1 genus and 1 species from both Euglenophyta and Chromophyta. The dominant phytoplankton species in the study areas were *Skeletonema costatum* (Greville) Cleve, *Aulacoseira granulata* (Ehrenberg) Simonsen, *Thalassiothrix frauenfeidii* Grunow, and *Thalassionema* *nitzschioides* Grunow. The phytoplankton abundance in the waters around Macau was between 46,607.14 and 1,355,000 cells/m^3^, with the highest abundance noted in station S8. Diatoms were the main contributor to phytoplankton abundance in station S8, accounting for 96.2% of the total abundance. Station S4 exhibited the lowest phytoplankton abundance of 46,607.1 cells/m^3^, with diatoms and Chromophytaaccounting for 58.6% and 29.9% of the total phytoplankton abundance, respectively. Biodiversity analysis results showed that the phytoplankton richness index was 1.18–3.61, the uniformity index was 0.24–0.78, and the Shannon–Wiener index was 0.94–3.41. Correlation analysis revealed that ammonia nitrogen was significantly negatively correlated with the phytoplankton richness, uniformity, and Shannon–Wiener indices. Nitrite nitrogen, nitrate nitrogen, inorganic nitrogen, salinity, turbidity, and pH were positively correlated with the phytoplankton evenness index and Shannon–Wiener index. Cluster and non-metric multidimensional scaling analyses demonstrated that the phytoplankton community structure in the waters around Macau could be divided into three groups, with *A. granulata*, *S. costatum*, *T. frauenfeidii*, *T. nitzschioides*, *Chaetoceros curvisetus* Cleve, and *Chaetoceros diadema* (Ehrenberg) Gran being predominant in different grouping communities (contribution% > 10%). Biota-Environment Stepwise Analysis (BIOENV) showed a significant correlation between the phytoplankton community and nitrite nitrogen content in the waters around Macau (correlation: 0.5544, Mantel test: statistic 0.4196, *p* = 0.009), which was consistent with the results of the canonical correspondence analysis.

## 1. Introduction

Phytoplankton are the main marine primary producer. As the basic link in the marine food web structure [1], phytoplankton play an important role in the material cycle and energy transformation of marine ecosystems [2]. A change in the phytoplankton community directly affects the succession of food webs in ecosystems, particularly the characteristics of the top consumer community [3], and the species composition, and community structure of phytoplankton are often used to characterize the water environment [4].

The community characteristics, distribution, and quantitative dynamics of dominant phytoplankton species are closely related to a variety of environmental factors [5]. Temperature is an important factor that affects the photosynthesis and respiration of phytoplankton, and it is closely related to the biological efficiency of phytoplankton [6,7,8]. An increase in temperature is related to changes in the standing stock of biomass of primary producers [7]. Salinity affects phytoplankton community composition and cell abundance [9]. For example, diatoms are usually dominant in low-salinity seawater, whereas dinoflagellates and cyanobacteria are more tolerant to high-salinity seawater. When seawater salinity exceeds 30 psu, the biomass of phytoplankton becomes negatively correlated with salinity [10]. In addition, light, water mass, current, monsoon conditions, and climate also affect the biomass, community structure, and distribution of phytoplankton [11,12,13].

At present, seawater warming and eutrophication are the two major environmental problems encountered by offshore ecosystems [14]. Diatoms and dinoflagellates are the two dominant phytoplankton groups [15] in offshore ecosystems, which cause the highest frequency of offshore algal blooms. Therefore, an analysis of the response of phytoplankton communities, especially diatoms and dinoflagellates, to seawater warming and eutrophication is very important to understand the impact of future environmental changes on the structure and function of offshore ecosystems [16]. Over the last 20 years, related studies have focused mainly on the quantitative dynamic relationship between nutrient input and phytoplankton, the evolutionary trends of dominant species and community structure of offshore phytoplankton under eutrophication [17,18,19], and the interaction of various stress factors to explore the effects of multiple factors on phytoplankton productivity and community structure [20]. The aims of this study were: (1) to investigate the response characteristics of phytoplankton species to environmental stressors; (2) to determine how these response characteristics are affected by these stressors, and (3) to assess the potential tradeoffs that may be involved, which could help to improve our conceptual understanding of the effects of multiple stressors on different phytoplankton populations [1]. Therefore, long-term monitoring of phytoplankton is crucial to understanding the coastal ecological environment and identifying the key environmental factors which affect it.

The waters around Macau are located in the channel between Lingdingyang Bay and Modaomen watercourse in the Pearl River Estuary and represent an important component of the Pearl River Estuary. Macau is one of the four central cities of the Guangdong–Hong Kong–Macau greater bay area. It has a population density of more than 20,000 inhabitants/km^2^, which makes it one of the most densely populated cities in the world, with a per capita GDP of about US$80,000, dominating the top of the ranking among the most economically developed regions in the world. With economic development and the discharge of pollutants such as domestic sewage and industrial wastewater, the ecological environment of Macau’s surrounding waters has been seriously damaged. According to a report on the environmental condition of Macau (2018), in the past 10 years, eutrophication along the coast of Macau has become serious, and masses of dead fish, black and odorous waters, and red tides frequently occur.

In the last decade, numerous studies have been performed on the ecology of phytoplankton in the Pearl River Estuary and its adjacent waters in China, and these have discussed the ecological characteristics of phytoplankton, mechanism of red tides, and grain size structure and spatial distribution of primary productivity [21,22,23,24,25,26,27,28,29]. However, there is still a lack of research on the community structure of phytoplankton and the relationship between phytoplankton and environmental factors in the waters around Macau.

In the present study, based on the survey data of phytoplankton in the waters around Macau, the phytoplankton community was analyzed by a multivariate statistical method, and the relationship between the phytoplankton community and environmental factors was evaluated. The primary objective of this study was to provide a reference to protect the biodiversity and resources in the waters around Macau and to provide basic data for the economic construction and offshore ecosystem management in the Guangdong–Hong Kong–Macau greater bay area.

## 2. Materials and Methods

### 2.1. Study Area

**Figure 1:**
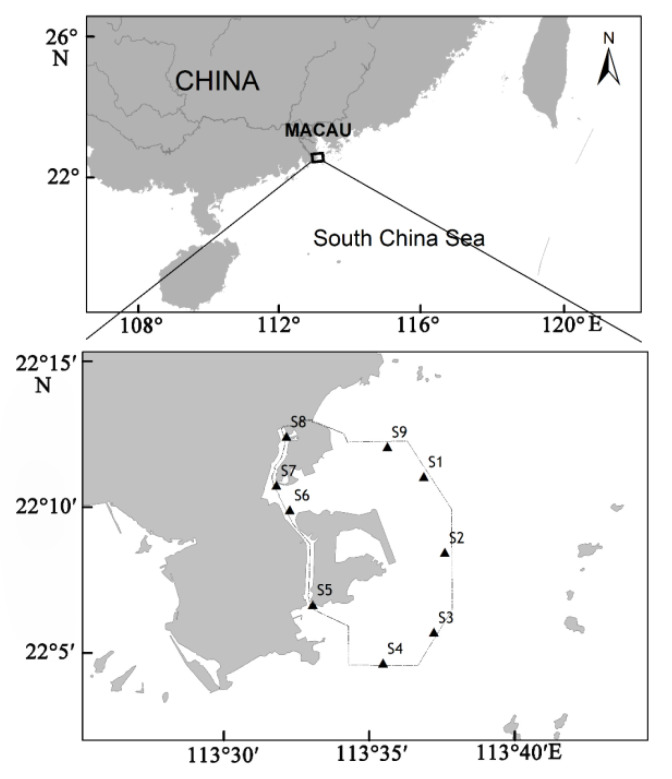
Location map of the sampling area.

The study was conducted in nine fixed locations in Macau waters from 30 December 2019 to 1 January 2020 (Figure 1).

### 2.2. Sample Collection

Phytoplankton sampling was performed in accordance with the specification for marine monitoring in China (GB/T 12763-2007). The sampling tool was a shallow-water type III plankton net (net length, 140 cm; net mouth inner diameter, 37 cm; net mouth area, 0.1 m^2^; and aperture, 0.077 mm). The samples were collected vertically from the bottom to the sea surface (Water depth between 3.5~7.1 m), stored in polyethylene (PE) bottles, fixed with Lugol’s Solution, and brought back to the laboratory.

### 2.3. Sample Processing and Analysis

The sample analysis was conducted according to the sedimentation concentration method [30]. In brief, after allowing the sample to stand for more than 48 h, the supernatant was siphoned and concentrated to a certain volume. Then, 0.1 mL of the concentrated supernatant was placed in the plankton counting box (10 × 10 mm) and the number of phytoplankton was counted under a light microscope (magnifying 400). Subsequently, the samples were subjected to species analysis and identification, and genera were determined when the species could not be identified. Environmental parameters (NH_3_-N, NO_2_-N, NO_3_-N, Inorganic-Nitrogen, Reactive phosphate) were ascertained in accordance with the specification for marine monitoring in China (GB/T 12763-2007), and the water temperature, salinity, and pH were measured using Multiparameter Sonde EXO_2_ (YSI Incorporated, Yellow Springs, OH, USA). The major forms of inorganic nitrogen are N_2_ gas, nitrate, nitrite, and ammonium. Reactive phosphate, commonly referred to as orthophosphate, is that portion of dissolved *P* that readily forms the phosphomolybdate blue complex.

### 2.4. Data Analysis

The biodiversity of phytoplankton was analyzed by the Shannon–Wiener index (*H′*) (1949):

(1) $$H^{\prime }=\sum_{i=1}^{S}p_{i}\log_{e}P_{i}$$

Species richness (*D*) was calculated as follows:

(2) $$D=\left(S-1\right)/\log_{2}N$$

Species evenness (*J′*) was determined as follows [31]:

(3) $$J^{\prime }=H^{\prime }/\log_{2}S$$

where *P_i_* is the ratio of the number of individuals of species *i* to the total number of individuals in the sample (*N_i_/N*), *N* is the total number of individuals of all species in the sample and *S* is the total number of species in the sample.

The dominance (*Y*) was calculated as follows [32]:

(4) $$Y=\left(n_{i}/N\right)\;f_{i}$$

where *N* is the total number of individuals of all species in the mud sample, *n_i_* is the number of individuals of species *i*, and *f_i_* is the frequency of the species at each station. A species was considered dominant when the species dominance *Y* > 0.02.

For community structure analysis, cluster analysis (Cluster) and non-metric multidimensional scaling (NMDS) analysis were employed to transform the square root of phytoplankton abundance data. Subsequently, Bray-Curtis similarity coefficients were calculated, a similarity matrix was established, and the average clustering method (average method) was used for clustering and ranking. Concurrently, based on Cluster results, SIMPROF (similarity profile) was utilized to test different groups, and species with a high contribution of SIMPER (similarity percentages) to the community were determined.

Canonical correspondence analysis (CCA) was used to understand the relationship between the phytoplankton community and environmental factors. Before the analysis, the phytoplankton data were transformed by square root, while environmental factors were transformed by *log*(*X* + 1). Subsequently, variance expansion factor analysis (variance inflation factor, VIF) was performed to exclude factors with minor contributions and determine the environmental factors that had a significant impact on the community. At the same time, Biota-Environment Stepwise Analysis (BIOENV) was employed to analyze the most fitting combination of environmental factors affecting the distribution of the biological communities, and data processing before the analysis was the same as that applied for CCA. A biological similarity matrix was constructed using Bray-Curtis similarity coefficients, and the environmental similarity matrix was created by Euclidean distance (Euclidean Distance). After BIOENV, the Mantel test was used to test the matrix correlation between phytoplankton abundance and environment parameters (used by the Spearman correlation test).

Phytoplankton diversity analysis, community structure visualization (Cluster and NMDS), and SIMPER were analyzed by PRIMER v6 software developed at the Plymouth Marine Laboratory [33,34], and the correlation between phytoplankton diversity and environmental factors was determined. The environmental data were transformed by “Hellinger” before analysis. The “vegan package” was employed for CCA, biological environment analysis, and the Mantel test, and “ggplot2” was utilized for the CCA triplot. Multivariate statistical analysis was conducted using R 4.0.2.

## 3. Results

### 3.1. Composition and Distribution of Phytoplankton

**Figure 2:**
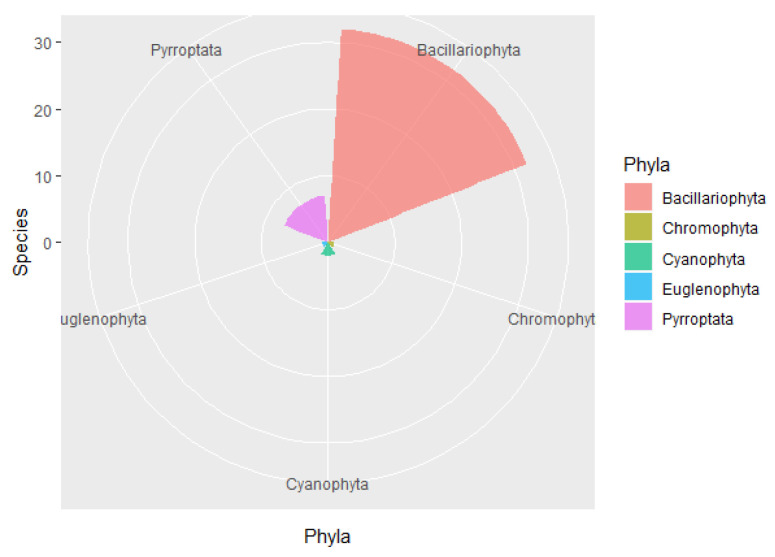
Phytoplankton species composition in the waters around Macau.

**Figure 3:**
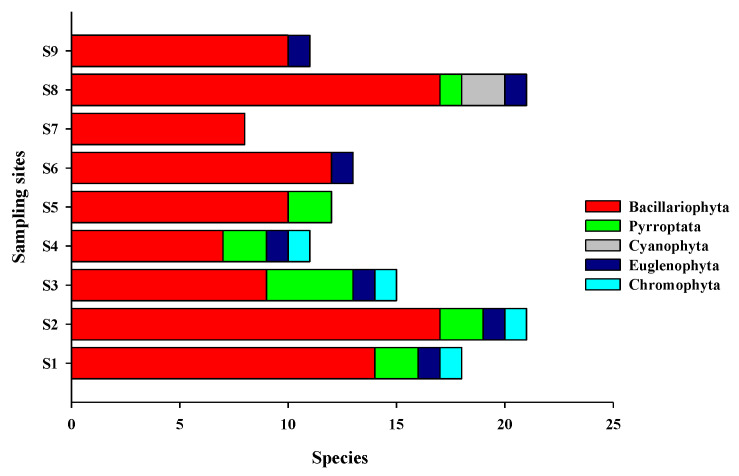
Phytoplankton species of different taxa detected in the different sampling sites.

A total of 43 species of phytoplankton belonging to 29 genera and 5 phyla were identified in the study area (Appendix A), including 32 species from 22 genera of Bacillariophyta, 7 species from 3 genera of Pyrrophyta, 2 species from 2 genera of Cyanophyta, 1 species from Euglenophyta, and 1 species from Chromophyta (Figure 2). In each survey station, diatoms accounted for 60–100% (average of 81.1%) of the total number of species and 58.60–100.00% (average of 359,172.90 cells/m^3^) of the total cell abundance, while dinoflagellates accounted for 4.76–18.18% (average of 9.66%) of the total number of species and 1.20–3.30% (average of 1927.60 cells/m^3^) of the total cell abundance (Figure 3). In stations S6, S7, and S9, dinoflagellates were undetected. The dominant phytoplankton species in the study areas were *Skeletonema costatum*, *Aulacoseira granulata*, *Thalassiothrix frauenfeidii*, and *Thalassionema nitzschioides* (Table 1).

The phytoplankton abundance in the study stations was 46,607.14–1,355,000 cells/m^3^, with the highest abundance noted in station S8. Diatoms were the predominant phytoplankton in station S8, accounting for 96.2% of the total phytoplankton abundance. In station S7, only diatoms were collected, which presented the second highest abundance of 466,250 cells/m^3^. The abundance of phytoplankton in station S4 was the lowest, reaching 46,607.1 cells/m^3^, and diatoms and Chromophyta accounted for 58.6% and 29.9% of the total phytoplankton abundance in this station, respectively (Table 2).

**Figure 4:**
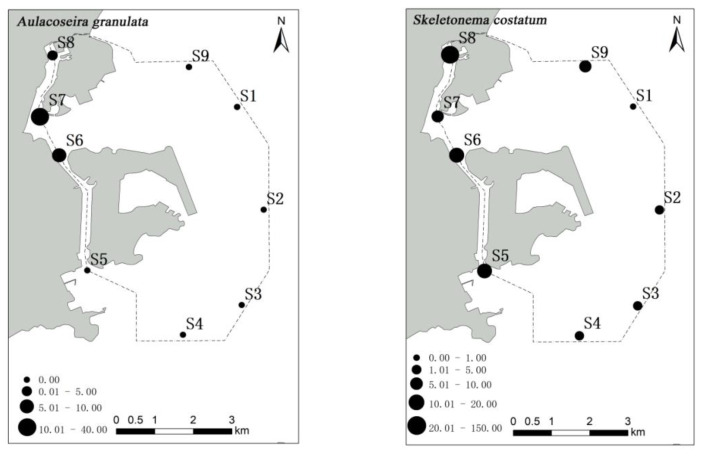

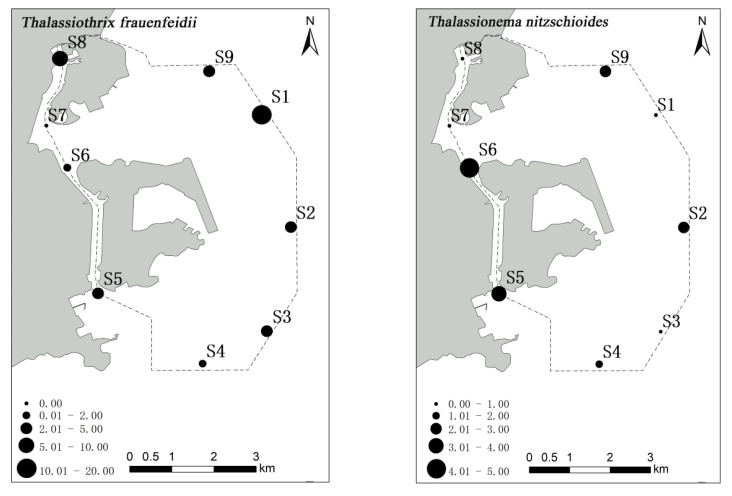
The spatial distribution of abundance (cell/m^3^) of phytoplankton dominant species in waters around Macau.

*A. granulata*, *S. costatum* and *T. nitzschioides* were found in high abundance mainly in the Qianshan and Shizimen waterways, while a high abundance of *T. frauenfeidii* was detected in the northeast sea area near Macau airport (Figure 4).

### 3.2. Diversity

The results of biodiversity indices are shown in Table 3. The richness index (*D′*) of phytoplankton ranged from 1.18 to 3.61, with maximum values noted in stations S2 and S3. The evenness index (*J′*) ranged from 0.24 to 0.78, with maximum values observed in stations S2 and S9. The Shannon–Wiener index (*H′*) was 0.94–3.41, with the highest values detected in stations S2 and S3. Analysis of the correlation among phytoplankton abundance, biodiversity index, and water environmental factors (such as ammonia nitrogen, nitrite nitrogen, nitrate nitrogen, inorganic nitrogen, active phosphate, salinity, turbidity, and pH) in the waters around Macau revealed the following results: phytoplankton abundance was negatively correlated with water turbidity; ammonia nitrogen was negatively correlated with the phytoplankton richness index, evenness index, and Shannon–Wiener index; and nitrite nitrogen, nitrate nitrogen, inorganic nitrogen, salinity, turbidity, and pH were positively correlated with the phytoplankton evenness index and Shannon–Wiener index (Table 4).

### 3.3. Community Structure

**Figure 5:**
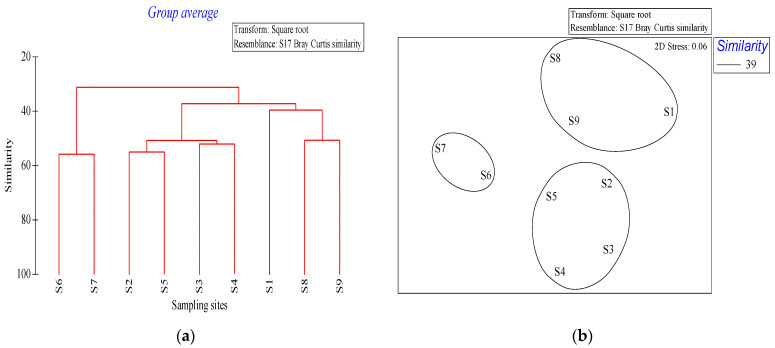
Cluster and NMDS analysis of phytoplankton structure in the waters around Macau examined in December 2019. (**a**) Cluster analysis of Bray-Curtis similarity indices of different sampling sites. (**b**) NMDS based on the Bray-Curtis similarity ordination (2D stress = 0.06). Ellipses around the clusters indicate the standard deviation of point scores based on sample-group clusters.

After square transformation of the phytoplankton abundance data, the results of Cluster and NMDS based on the Bray–Curtis similarity matrix were found to be consistent (Figure 5). The nine stations could be divided into three groups at the 95% dissimilarity level (dissimilarity level) based on SIMPROF test results. Group A consisted of stations S6 and S7; group B comprised stations S2, S3, S4, and S5; and group C included stations S1, S8, and S9 (Figure 4).

According to the abundance matrix of phytoplankton in the survey site, the average similarity contribution rate of different phytoplankton species groups was analyzed by SIMPER, and the species presenting the specific characteristics of the community (species with an average similarity contribution rate of >10%) were selected (Table 5). The results showed that the average intragroup similarity of group A was 55.8%, with *A. granulata* and *S. costatum* significantly contributing to intra-group similarity. The average intra-group similarity of group B was 51.7%, with *S. costatum*, *T. frauenfeidii*, and *T. nitzschioides* significantly contributing to intra-group similarity. The average intra-group similarity of group C was 43.24%, with *T. frauenfeidii, Chaetoceros curvisetus, C. diadema,* and *S. costatum* significantly contributing to intra-group similarity.

### 3.4. Relationship between the Phytoplankton Community and Environmental Variables

**Figure 6:**
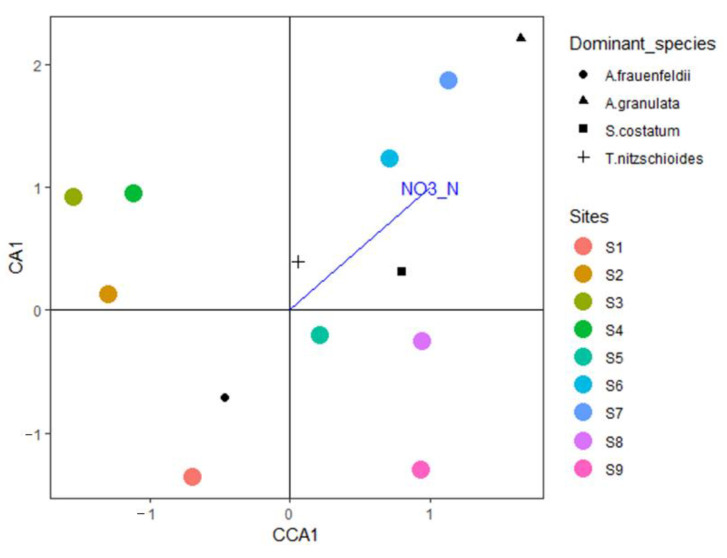
CCA of the relationship between environmental parameters and phytoplankton species abundance in the waters around Macau examined in December 2019.

BIOENV analysis of the phytoplankton community and eight physical and chemical factors in the waters around Macau showed that nitrite nitrogen (correlation: 0.5544) was the environmental variable that had the greatest impact on the phytoplankton species matrix (Mantel test: statistic 0.4196, *p* = 0.009) (Table 6). VIF analysis, excluding the insignificant factors, revealed that nitrite nitrogen had a significant effect on the phytoplankton community in the waters around Macau (VIF = 82.28, F = 2.11, *p* < 0.01) (Figure 6).

## 4. Discussion

The results of the present study indicated that diatoms are the major phytoplankton species in the waters around Macau, which is consistent with data from the inshore waters of the northern South China Sea [22,23,24,35,36]. The phytoplankton species that can thrive under normal and warm temperature conditions were dominant and showed obvious tropical and subtropical characteristics [22,23]. The surveyed waters are affected by fresh water and tidal currents. The phytoplankton species could be divided into different ecological types, such as low-salt nearshore, widespread offshore, and high-salt offshore, and this classification is consistent with that reported by Jia et al. (2019) [21]. For example, *S. costatum* is a nearshore low-salt phytoplankton species, whereas *Rhizosolenia setigera* Brightwell, *T. nitzschioides, Ditylum brightwellii, T. frauenfeidii*, etc., are widespread offshore phytoplankton species with a wide range of adaptations to temperature and salinity. *Rhizosolenia styliformis* Brightwell and *C. siamense* Ostenfeld are species that can adapt to high salinities and varying temperatures and could have been transported to the study area from the open sea by tidal currents. The phytoplankton taxa predominantly detected in the study area belonged to the widely distributed offshore group, and these results are consistent with those reported in previous studies [28,37].

The dominant phytoplankton species in the waters around Macau were *S. costatum*, *A. granulata, T. frauenfeidii*, and *T. nitzschioides*, similar to those identified in previous studies [28,38,39]. *S. costatum* is a red tide alga that is abundant in the coastal waters of Guangdong [40]. Between the years 2000 and 2009, 36 red tides were recorded in the waters near the Pearl River Estuary. Diatoms, mainly *S. costatum*, are the predominant red tide algae [41,42], especially in the high water season [23], and numerous studies have reported similar findings in other waters, such as the East China Sea [43]. While *S. costatum* does not produce phycotoxins, its major effect on the marine ecosystem is oxygen depletion caused by the appearance of red tides [44]. *A. granulata* is a universal phytoplankton species, which is particularly dominant in summer and in medium-eutrophic water. It is often used as an indicator of eutrophication and polluted water [45,46]. *T. frauenfeidii* is a widespread phytoplankton species, which can also cause red tides and is one of the dominant phytoplankton species in the coastal waters, bays, and estuaries of China [47]. As a common diatom found worldwide, *T. nitzschioides* is distributed in all major seas and shallow seas from the equator to high latitudes, except in the southern and arctic regions [48]. Furthermore, *T. nitzschioides* has been reported to be a good indicator of change in seawater temperature [49].

In marine phytoplankton studies, the diversity index is a commonly used biodiversity measurement tool that can better reflect the attributes of the phytoplankton community and its relationship with the environment [50]. The results of the present study showed that the spatial distribution of phytoplankton biodiversity in the waters around Macau is much lower than that in the open sea, suggesting that the phytoplankton community in the waterway area is significantly disturbed. Frequent interference by human activities (such as shipping and flood discharge) and eutrophication are the main factors that affect the diversity of phytoplankton communities in waterways. The results of the present study are consistent with the phytoplankton biodiversity observed in the adjacent waters of the Pearl River Estuary [22,23,24,28,39]. Correlation analysis between the diversity index and environmental factors revealed a significant negative correlation between phytoplankton abundance and turbidity, and a significant positive correlation between phytoplankton biodiversity and nitrogen nutrients in the water. The instability of the water body in the waterway area is influenced by multiple stresses, such as waterway transportation and drainage, which affects the growth of phytoplankton on the surface of the water. Furthermore, land source discharge and shipping disturbance increase the turbidity of the water body, which affects the absorption of light by plants. The light restriction also decreases phytoplankton abundance. The differences in nutrient levels may potentially limit the absorption and utilization of nutrients by phytoplankton and regulate the phytoplankton population structure. While the growth of some phytoplankton species could be restricted by the lack of certain nutrients, other phytoplankton species with relatively low demand for these nutrients can rapidly grow and become dominant, resulting in simplification of the species composition and a decrease in biodiversity. In the present study, the low Shannon–Wiener index and moderate species richness reflected the uneven distribution of the phytoplankton species, with diatoms dominating the phytoplankton community in the study sites.

Spatial changes in phytoplankton communities are an important component of many aspects of ecology, including the maintenance of species diversity and community stability [51,52]. The spatial distribution of phytoplankton communities is mainly caused by the heterogeneity of the environment on the spatial scale [53]. In the present study, a high abundance of phytoplankton was observed in stations S7 and S8 of the Qianshan waterway and station S6 of the Shizimen waterway. The abundance of *S. costatum* in stations S8 and S7 reached 1380 and 289 cells/m^3^, respectively. The distribution of *S. costatum* is negatively correlated with salinity and transparency [54]. In the waters around Macau, the inflow of fresh water from the Pearl River decreases the salinity and transparency, making it suitable for the growth of *S. costatum*. Similar results have also been noted in the Yangtze Estuary [55]. Some studies have shown that *S. costatum* can grow better in water with a high N/P ratio, such as the Pearl River Estuary. Some studies have shown that eutrophic water bodies are more suitable for *A. granulata* growth, and that the mixed stirring environment is more conducive to maintaining *A. granulata* in the true light layer [56]. In the present study, *A. granulata* was distributed in stations S6 and S8, but its abundance was much lower than that observed in S7. Furthermore, a high number of *T. frauenfeidii* (221 cells/m^3^) was observed in station S1, which may be related to the temperature, water depth, salinity, or nutrient concentration [57,58].

The NMDS results revealed that the phytoplankton communities in the waters around Macau could be mainly divided into the *A. granulata*—*S. costatum*, *S. costatum—T. frauenfeidii—T. nitzschioides*, and *T. frauenfeidii—C. curvisetus—C. diadema*—*S. costatum* communities. The *A. granulata—S. costatum* community was detected in the Qianshan waterway, which has a high nutrient content. The Qianshan waterway is affected by the freshwater inflow from the Pearl River, resulting in relatively low salinity, which promotes *S. costatum* growth [59]. The salinity of stations S6 and S7 was 5.14–7.6 psu, Huo et al. (2001) reported 10–20 psu was the optimal salinity range for *S. costatum* growth [60]. The *S. costatum—T. frauenfeidii—T. nitzschioides* community was detected in the waters southeast of Macau, mainly owing to the ecological characteristics of these species. *Thalassiothrix frauenfeidii* and *T. nitzschioides* can tolerate a wide range of temperatures and salinities, and *T. nitzschioides* populations arranged in chain. The *T. frauenfeidii—C. curvisetus—C. diadema—S. costatum* community was detected in the northeast of the survey waters. *C. diadema* is a coastal species distributed from the northern temperate zone to the Arctic region. It has adapted to a wide range of temperatures and salinities and is widely distributed around the world. In this study, *C*. *diadema* was detected in the offshore stations S1 and S9. *Chaetoceros curvisetus* Cleve is a eurythermic coastal species, which presented the second highest cell abundance; however, its distribution range was small, indicating its narrow ecological adaptation range.

Phytoplankton must absorb inorganic nutrients (mainly nitrate, nitrite, silicate, and phosphate) from the water body for their own growth and reproduction. Therefore, the nutrient concentration in a water body is the primary factor affecting phytoplankton communities [61]. In the northern part of the South China Sea, nutrient concentration is the primary limiting factor for phytoplankton growth. Owing to terrestrial nutrient supplementation, the Pearl River Estuary and coastal areas do not usually face nutrient limitations. As a result, phytoplankton cell abundance is higher in these areas, when compared with those in the shelf and open sea areas [62]. Long et al. analyzed the surface water of the northern South China Sea and concluded that the growth of phytoplankton and nitrite content in the northern South China Sea are more closely related than other nutrient factors [63]. Song et al. (2013) suggested that Hong Kong’s coastal waters are more likely to be restricted by N content in various seasons [64]. Considering the minimum nutrient threshold, winter nutrients do not restrict the growth of phytoplankton. In the present study, the CCA of the phytoplankton community and environmental factors revealed a significant correlation between nitrite nitrogen and phytoplankton abundance. The CCA triplot indicated that *S. costatum*, *A. granulata*, and *T. nitzschioides* growth, especially *S. costatum* growth, is closely related to nitrite nitrogen content. Previous studies have shown that different N/P ratios in the environment affect the specific growth rate and cell state of *S. costatum*, and that the N content restricts growth more than P content. *S. costatum* growth is significantly correlated with total nitrogen and dissolved inorganic nitrogen contents in Baltic coastal waters [65]; however, Lagus et al. indicated that *S. costatum* growth was mainly restricted by P content. *A. granulata* is known to adapt to low-salinity conditions [66], and its distribution is mainly determined by the nutrient distribution pattern, as well as salinity [67]. Furthermore, previous studies have demonstrated that the growth of *T. nitzschioides* is mainly controlled by temperature and nutrient concentration. *T. nitzschioides* is considered to be a good indicator of changes in the nutrient concentration in the South-North Sea [68].

In coastal areas where river runoff injects a high level of nutrients, a continuous influx of ammonium-utilizing, nitrite-utilizing, and nitrifying microorganisms can cause competition and restriction in the phytoplankton population and maintain a temporary coexisting population of nitrifying bacteria [69]. The coastal phytoplankton communities with a high degree of eutrophication tend to be of smaller size [70,71], and small diatoms (such as *Skeletonema* sp. and *Chaetoceros* sp.) may be well represented in areas with a high degree of eutrophication. Nevertheless, further studies are needed to perform long-term monitoring of phytoplankton communities to determine the response of phytoplankton to multiple stresses in the coastal waters of Macau.

## 5. Conclusions

The phytoplankton community in the waters of Macau was mainly composed of diatoms and dinoflagellates, based on the investigation of the phytoplankton community and environmental factors in January 2020. Diatoms were the dominant group, accounting for 81.10%. Among the environmental factors, only ammonia nitrogen was negatively correlated with phytoplankton diversity, and inorganic nitrogen content had a significant effect on phytoplankton community structure.

## Figures and Tables

**Table 1:** Dominant phytoplankton species collected by net from the waters around Macau.

Dominant Species	Percentage of Abundance	Frequency	Dominance (*Y*)
*Skeletonema costatum*	51.31%	0.89	0.46
*Thalassiothrix frauenfeidii*	13.23%	0.89	0.12
*Aulacoseira granulata*	11.14%	0.33	0.04
*Thalassionema nitzschioides*	4.04%	0.78	0.03

**Table 2:** Spatial distribution of phytoplankton abundance in the waters around Macau (cells/m^3^).

Sampling Sites	Depth (m)	Bacillariophyta	Pyrrophyta	Cyanophyta	Euglenophyta	Chromophyta
S1	6.0	300,750.00	4500.00	0.00	3000.00	4500.00
S2	6.9	165,942.03	2173.91	0.00	7246.38	9420.29
S3	7.1	64,084.51	2816.90	0.00	10,563.38	7042.25
S4	5.6	27,321.43	1071.43	0.00	4285.71	13,928.57
S5	3.5	274,285.71	4285.71	0.00	0.00	0.00
S6	4.8	303,125.00	0.00	0.00	1041.67	0.00
S7	4.0	466,250.00	0.00	0.00	0.00	0.00
S8	3.6	1,342,500.00	2500.00	2500.00	7500.00	0.00
S9	4.7	288,297.87	0.00	0.00	1063.83	0.00

**Table 3:** Biodiversity indices of phytoplankton communities in the waters around Macau.

Sampling Sites	*S*	*D′*	*J′*	*H′*
S1	18	2.82	0.61	2.53
S2	21	3.61	0.78	3.41
S3	15	2.92	0.71	2.76
S4	11	2.24	0.77	2.65
S5	12	2.09	0.63	2.27
S6	13	2.11	0.54	2.00
S7	8	1.18	0.31	0.94
S8	21	2.71	0.24	1.05
S9	11	1.78	0.78	2.71

**Table 4:** Correlation coefficient of richness index (*D′*), Pielou evenness index (*J′*), Shannon–Wiener index (*H′*), depth, abundance, ammonia nitrogen, nitrite nitrogen, nitrate nitrogen, inorganic nitrogen, reactive phosphate, salinity, turbidity, and pH of phytoplankton in the waters around Macau examined in December 2019.

	*D′*	*J′*	*H′*	Abundance	NH_3_-N	NO_2_-N	NO_3_-N	Inorganic-N	Reactive Phosphate	Salinity	Turbidity	pH
*D′*	1											
*J′*	0.33	1										
*H′*	0.59	0.95	1									
abundance	0.08	−0.78	−0.64	1								
NH_3_-N	−0.71 *	−0.77 *	−0.88 **	0.53	1							
NO_2_-N	0.62	0.6 *	0.75 *	−0.29	−0.84	1						
NO_3_-N	0.19	0.75 *	0.72 *	−0.39	−0.62	0.77	1					
Inorganic-N	0.18	0.73 *	0.7	−0.37	−0.6	0.77	1	1				
Reactive phosphate	−0.22	0.47	0.29	−0.26	−0.15	0.02	0.58	0.57	1			
Salinity	0.63	0.83 **	0.9 **	−0.6	−0.98	0.82	0.68	0.66	0.27	1		
Turbidity	0.51	0.85 **	0.88 **	−0.76 *	−0.77	0.52	0.39	0.36	0.03	0.78	1	
pH	0.63	0.81 **	0.88 **	−0.55	−0.98	0.85	0.72	0.7	0.28	1	0.73	1

* Significance level: *p* < 0.05. ** Significance level: *p* < 0.01.

**Table 5:** Similarity analysis of the phytoplankton community composition in the waters around Macau examined in December 2019.

Groups	Species	Av. Abund	Contrib %	Cum. %
A	*Aulacoseira granulata*	1313.68	44.3	44.3
	*Skeletonema costatum*	1020.38	41.37	85.67
B	*Skeletonema costatum*	631.92	24.27	24.27
	*Thalassiothrix frauenfeidii*	474.13	14.86	39.14
	*Thalassionema nitzschioides*	376.77	10.78	49.91
C	*Thalassiothrix frauenfeidii*	1046.19	24.91	24.91
	*Chaetoceros curvisetus*	540.54	17.99	42.9
	*Chaetoceros diadema*	520.53	12.55	55.45
	*Skeletonema costatum*	1558	10.65	66.1

**Table 6:** Results from the BIO-ENV procedure showing the best overall combination of environmental parameters in the waters around Macau examined in December 2019.

Parameters	Size	Correlation
NO_2_-N	1	0.5544
NO_2_-N, Salinity	2	0.5302
NO_2_-N, Salinity, pH	3	0.5302
NO_2_-N, DIN, Salinity, pH	4	0.5158
NO_2_-N, NO_3_-N, DIN, Salinity, pH	5	0.5022
NO_2_-N, NO_3_-N, DIN, DIP, Salinity, Turbidity	6	0.4831
NO_2_-N, NO_3_-N, DIN, DIP, Salinity, Turbidity, pH	7	0.4698
NH_4_, NO_2_-N, NO_3_-N, DIN, DIP, Salinity, Turbidity, pH	8	0.4196

## Data Availability

Not applicable.

## Appendix A

**Table A1:** List of phytoplankton in the waters of Macao.

Phyla	Species
Bacillariophyta	*Stephanopyxis palmeriana* (Greville) Grunow, 1844
	*Stephanopyxis turris* (Greville) Ralfs, 1861
	*Odontella sinensis* (Greville) Grunow, 1884
	*Eucampia zodiacus* Ehrenberg, 1839
	*Ditylum brightwellii* (T.West) Grunow, 1885
	*Gyrosigma* sp.
	*Skeletonema costatum* (Greville) Cleve 1900
	*Hemiaulus sinensis* Greville
	*Hemiaulus hauckii* Grunow ex Van Heurck, 1882
	*Melosira granulata* (Ehrenberg) Simonsen
	*Thalassiosiria rotula* Meunier, 1910
	*Planktoniella sol* (Wallich) Schütt, 1892
	*Corehtron pelagicum* Brun, 1891
	*Nitzschia* sp.
	*Nitzschia longissima* (Brébisson) Ralfs, 1861
	*Nitzschia lorenziana* Grunow, 1879
	*Rhizosolenia setigera* Brightwell, 1858
	*Pseudo-nitzschia pungens* (Grunow ex Cleve) G.R.Hasle, 1993
	*Bacilaria paxillifera* (O.F.Müller) T.Marsson, 1901
	*Chaetoceros decipiens* Cleve, 1873
	*Chaetoceros diadema* (Ehrenberg) Gran, 1897
	*Chaetoceros curvisetus* Cleve, 1889
	*Chaetoceros siamense* Ostenfeld, 1902
	*Chaetoceros tortissimus* Gran, 1900
	*Chaetoceros wighamii* Brightwell, 1856
	*Coscinodiscus* sp.
	*Coscinodiscus wailesii* Gran and Angst, 1931
	*Thalassiothrix frauenfeldii* (Grunow) Grunow, 1880
	*Thalassiothrix nitzschioides* (Grunow) Grunow, 1881
	*Synedra* sp.
	*Fragilaria* sp.
	*Surirella robusta* Ehrenb., 1841
Pyrrophyta	*Ceratium tripos* (O.F.Müller) Nitzsch, 1817
	*Ceratium extensum* (Gourret) Cleve-Euler, 1900
	*Ceratium extensum* f. *strictum* Nielsen, 1957
	*Ceratium massiliense* (Gourret) Karsten, 1906
	*Ceratium furca* (Ehrenberg) Claparède and Lachmann, 1859
	*Dinophysis caudata* Saville-Kent, 1881
	*Protoperidinium oceanicum* (VanHöffen, 1897) Balech, 1974
Cyanophyta	*Oscillatoria* sp.
	*Spirulina* sp.
Euglenophyta	*Euglena ehrenbergii* G.A.Klebs
Chromophyta	*Phaeocystis globosa* Scherffel, 1899
